# Supplementary material for: Change in Collagen Fibril Diameter Distribution of Bovine Anterior Cruciate Ligament upon Injury Can Be Mimicked in a Nanostructured Scaffold
Source: Molecules. 2021 Feb 24;26(5):1204. doi: 10.3390/molecules26051204 (PMC7956598; doi:10.3390/molecules26051204)
Supplement: Supplementary file 1 [file molecules-26-01204-s001.pdf]

Table S1. Data for mechanical characterization of ACL

	Thickness mm	Width mm	Area (mm^2)	Area (m^2)	Length (mm)	Max Load (N)	Max Load (kN)	Ultimate Stress (Mpa)	Ultimate Strain (mm)	Ultimate Strain (%)	Strain @ Break (mm)	Strain @ Break (%)	Modulus (MPa)	Strain Energy Density (MPa)
**Average**	**5.2**	**13.4**	**70.9**	**0.0**	**35.0**	**2731.4**	**2.7**	**41.0**	**21.4**	**61.0**	**46.8**	**133.6**	**0.8**	**1140.1**
**STD**	**0.7**	**2.7**	**20.1**	**0.0**	**0.9**	**484.4**	**0.5**	**13.3**	**4.3**	**10.8**	**7.5**	**19.3**	**0.3**	**288.5**
**Outliar Upper**	**6.4**	**17.9**	**104.0**	**0.0**	**36.5**	**3530.6**	**3.5**	**62.8**	**28.5**	**78.8**	**59.1**	**165.5**	**1.3**	**1616.2**
**Outliar lower**	**4.1**	**8.9**	**37.8**	**0.0**	**33.4**	**1932.2**	**1.9**	**19.1**	**14.3**	**43.1**	**34.5**	**101.7**	**0.3**	**664.1**
Joint#2	4.51	15.65	70.60	0.0000706	34.48	1925.0	1.93	27.27	19.00	55.10	50.00	145.01	0.66	733
Joint#3	6.15	15.96	98.10	0.0000981	36.13	2900.0	2.90	29.56	27.00	74.72	57.00	157.75	0.48	967
Joint#4	5.77	14.14	81.58	0.0000816	34.98	3225.0	3.23	39.53	24.00	68.61	48.00	137.22	0.70	1207
Joint#5	4.80	11.83	56.82	0.0000568	35.59	2857.0	2.86	50.28	21.00	59.01	40.00	112.40	0.95	1373
Joint#6	4.96	9.53	47.25	0.0000473	33.70	2750.0	2.75	58.20	16.00	47.48	39.00	115.73	1.28	1421

Table S2. Data for mechanical characterization of PCL scaffolds

	Thickness mm	Width mm	Area (mm^2)	Area (m^2)	Length (mm)	Max Load (N)	Max Load (kN)	Ultimate Stress (Mpa)	Ultimate Strain (mm)	Ultimate Strain (%)	Strain @ Break (mm)	Strain @ Break (%)	Modulus (MPa)	Strain Energy Density (MPa)
**Average-Aligned**	**0.077**	**10.00**	**0.77**	**0.00**	**33.42**	**2.93**	**0.0029**	**3.98**	**12.92**	**31.38**	**16.80**	**40.80**	**1.37**	**123.58**
**STD**	**0.025**	**0.00**	**0.25**	**0.00**	**3.72**	**0.09**	**0.0001**	**1.36**	**0.84**	**2.03**	**0.53**	**1.29**	**0.52**	**43.27**
**Outlier Upper**	**0.117**	**10.00**	**1.17**	**0.00**	**39.56**	**3.08**	**0.0031**	**6.22**	**14.30**	**34.74**	**17.68**	**42.94**	**2.22**	**194.98**
**Outlier lower**	**0.037**	**10.00**	**0.37**	**0.00**	**27.28**	**2.78**	**0.0028**	**1.74**	**11.54**	**28.03**	**15.92**	**38.67**	**0.51**	**52.19**
**Average-Unaligned**	**0.034**	**10.00**	**0.34**	**0.00**	**29.72**	**0.28**	**0.0003**	**0.88**	**6.26**	**17.35**	**7.42**	**20.47**	**0.15**	**11.31**
**STD**	**0.005**	**0.00**	**0.05**	**0.00**	**9.24**	**0.11**	**0.0001**	**0.26**	**0.83**	**3.15**	**0.13**	**1.30**	**0.10**	**3.86**
**Outlier Upper**	**0.043**	**10.00**	**0.43**	**0.00**	**44.97**	**0.46**	**0.0005**	**1.30**	**7.62**	**22.55**	**7.63**	**22.61**	**0.32**	**17.68**
**Outlier lower**	**0.025**	**10.00**	**0.25**	**0.00**	**14.47**	**0.09**	**0.0001**	**0.45**	**4.90**	**12.15**	**7.20**	**18.32**	**-0.02**	**4.94**
